# Exploring *Blastocystis* genetic diversity in rural schoolchildren from Colombia using next-generation amplicon sequencing reveals significant associations between contact with animals and infection risk

**DOI:** 10.1007/s00436-023-07841-3

**Published:** 2023-05-09

**Authors:** Paula C. Hernández, Jenny G. Maloney, Aleksey Molokin, Nadja S. George, Liliana Morales, Jacqueline Chaparro-Olaya, Monica Santin

**Affiliations:** 1 Laboratorio de Parasitología Molecular, Vicerrectoría de Investigaciones, Universidad El Bosque, Bogotá, Colombia; 2 Environmental Microbial and Food Safety Laboratory, Agricultural Research Service, United States Department of Agriculture, Beltsville, MD 20705 USA

**Keywords:** *Blastocystis*, Colombia, Subtypes, Next-generation amplicon sequencing, Risk factors, Zoonotic transmission

## Abstract

*Blastocystis* is a common intestinal protist with a global distribution in humans and many other animals. Yet, the status of *Blastocystis* as a pathogen, the risk factors associated with its transmission, and its zoonotic potential remain ill-defined. Here, we explored subtype (ST) diversity and potential risk factors for *Blastocystis* infection in 98 children from Apulo, Colombia. Samples were screened for *Blastocystis* via PCR, and ST identification was performed through next-generation amplicon sequencing (NGS). Associations between the presence of *Blastocystis* and individual STs and sociodemographic variables were assessed via logistic regression analyses. Seventy-one samples (72.4%) were *Blastocystis*-positive, and NGS revealed the presence of five STs (ST1-ST5). ST1, ST2, and ST3 were common and observed in nearly equal proportions (~ 40%), while samples with ST4 (1.4%) and ST5 (5.6%) were comparatively rare. The presence of mixed STs in the same sample was also common (28.2%). Comparisons among children within the same household identified that shared ST profiles were common, but diversity within family units was also observed. Logistic regression analyses returned significant associations between the presence of *Blastocystis*, individual subtypes, or mixed subtypes for several variables. Intriguingly, the presence of animals was one of the most common significant associations. Taken together, these data represent an important step forward in understanding both the potential routes and risk factors that may influence *Blastocystis* transmission and will be useful in shaping future studies which seek to clarify the relationships between STs, pathogenicity, and zoonotic transmission.

**Supplementary Information:**

The online version contains supplementary material available at 10.1007/s00436-023-07841-3.

## Introduction

Human populations in developing countries frequently suffer from intestinal disease with intestinal parasite infections being one of the most common causes of disease (Kantzanou et al. 2021; Pazmiño et al. 2022). Intestinal parasite infections are major public health concerns, especially in pediatric populations in low- and middle-income countries where chronic infections could add to worsening illness and undernutrition as well as having long-term health implications (George et al. 2018; Hanieh et al. 2021; Deka et al. 2022). *Blastocystis* is one of the most prevalent protists in developing and developed countries, and it is estimated to colonize more than one billion people worldwide (El Safadi et al. 2014; Andersen and Stensvold 2016; Botero-Garcés et al. 2021). In Colombia, the reported prevalence of *Blastocystis* in human population studies has ranged from 40 to 70% (e.g., Hernández et al. 2019; Villamizar et al. 2019; Osorio-Pulgarin et al. 2021). In the pediatric population, the most recent ‘Encuesta Nacional de Parasitismo Intestinal en Población Escolar Colombiana (ENPI) 2015’ conducted by the Ministry of Health and Social Protection of Colombia between 2012 and 2014 identified *Blastocystis* as the most prevalent parasite in children (60%) (Ministerio de Salud 2015).

*Blastocystis* infects a wide range of hosts, including humans and animals (food-producing animals, companion animals, and wildlife) (Hublin et al. 2021). An extensive genetic diversity has been observed in *Blastocystis* isolates obtained from avian and mammalian hosts based on polymorphisms in the small subunit ribosomal RNA (SSU rRNA) gene. Based on that polymorphism, there are currently 34 distinct genetic variants, designated as subtypes (STs), meeting the requirements for subtype designation (ST1–ST17, ST21, and ST23–ST38) (Stensvold and Clark 2020; Higuera et al. 2021; Maloney and Santín 2021; Maloney et al. 2021a, 2022; Baek et al. 2022). *Blastocystis* subtypes do not appear to have a strong host specificity. Thirteen subtypes have been reported in both human and other animal hosts (ST1-ST8, ST10, ST12, ST14, ST16, and ST23) suggesting the potential for zoonotic transmission of those subtype (Alfellani et al. 2013; Jinatham et al. 2021; Khaled et al. 2021, 2020; Jinatham et al. 2021; Osorio-Pulgarin et al. 2021; Ramírez et al. 2016). In Colombia, *Blastocystis* subtyping studies have been conducted in different regions of the country, have included human and animal hosts, and have identified overlapping STs in multiple host species indicating potential zoonotic transmission (e.g., Ramírez et al 2014, 2016, 2017; Sánchez et al. 2017; Higuera et al 2020, 2021; Baek et al. 2022). Thus, to understand zoonotic transmission and public health significance of this parasite, it is crucial to accurately identify *Blastocystis* STs present in samples.

The value of the use of next-generation amplicon sequencing (NGS) to accurately identify *Blastocystis* subtypes and unravel mixed ST infections has been documented in human and animal populations (Maloney et al. 2019, 2021b; Rojas-Velázquez et al. 2019; Sarzhanov et al. 2021). However, only a single study in Colombia has been conducted using NGS in humans to examine *Blastocystis* subtype diversity (Vega et al. 2021). The study evaluated gut microbiota profiles with co-occurrence of *Clostridioides difficile* and *Blastocystis* in diarrheic patients and noted that mixed infections were common (16/31; 51.6%). In the present study, *Blastocystis* subtype diversity was assessed in schoolchildren in a rural region of Colombia using a next-generation amplicon sequencing strategy to explore mixed ST infections. Additionally, logistic regression analysis was used to evaluate associations between *Blastocystis* infection and socioeconomic and demographic variables within this population.

## Material and methods

### Study population and ethical statement

The present study was retrospectively carried out on 98 fecal samples obtained in a cross-sectional study conducted in school-aged children (4 to 16 years old) who attended three rural schools of Apulo, (Department of Cundinamarca, Colombia) to study intestinal parasitic infections in 2017 (Hernández et al. 2019). The study adhered to principles in the Declaration of Helsinki and guidelines of the Ministry of Health of Colombia for procedures involving human subjects and the ethics committee of Universidad El Bosque approved the study (#007–2017). This study was minimal risk. Written informed consent was obtained from the participants’ parents or guardians.

### Study population and sample collection

The sample collection was previously fully described (Hernández et al. 2019). Briefly, each participant provided a single stool sample collected in the early morning at their schools. Fecal samples were placed in capped containers with wide mouths and transported in cold containers to the Apulo public health center.

DNA was extracted from approximately 200 µl of concentrated stool sample (Mini-Parasep SF, DiaSys Ltd., Berkshire, England) using the QIAamp DNA Stool Mini Kit (Qiagen, Germany) according to the manufacturer’s instructions with minor modifications, as previously described (Hernández et al. 2019). DNA was stored at -20 °C until molecular analyses were performed.

### Molecular detection, NGS amplicon library preparation, and bioinformatic analysis

Next-generation amplicon sequencing libraries were prepared as previously described (Maloney et al. 2019). Briefly, all samples were screened by PCR using primers ILMN_Blast505_532F and ILMN_Blast998_1017R. These primers amplify a ca. 500 bp fragment of the *SSU* rRNA gene and are identical to Blast505_532F/Blast998_1017R (Santin et al. 2011), except for containing the Illumina overhang adapter sequences. PCR products were analyzed using a QIAxcel (Qiagen, Valencia, CA, USA). A final pooled library concentration of 8 pM with 20% PhiX control was sequenced using Illumina MiSeq 600 cycle v3 chemistry (Illumina, San Diego, CA, USA). Paired end reads were processed and analyzed with an in-house pipeline that uses the BBTools package v38.22 (Bushnell 2014), VSEARCH v2.8.0 (Rognes et al. 2016), and BLAST + 2.7.1. After removing singletons, clustering and the assignment of centroid sequences to operational taxonomic units (OTU) was performed within each sample at a 98% identity threshold. Only those OTUs with a minimum of 100 sequences were retained. Raw FASTQ files were submitted to NCBI’s sequence read archive under project PRJNA896134 and accession numbers SRR22105641- SRR22105683. The nucleotide sequences obtained in this study have been deposited in GenBank under the accession numbers OP725923-OP725977.

### Demographic/sociodemographic variables and logistic regression analysis

At the time of providing the samples, a questionnaire was administered to collect information on the following variables: gender (male or female), age (range 4–16 years), school (Naranjalito “T”, Naranjal “N”, or Pantanos “P”), monthly income in Colombian Peso (≤ 500 k, 500 k to 1000 k, ≥ 1000 k), number of people per household (≤ 3, 4 to 6, or ≥ 7 people per house), type of flooring (dirt or cement); aqueduct service (yes or no), toilet location (inside, peri-domestic, or outside the house), dogs enter the house (yes or no), cats enter the house (yes or no), chickens enter the house (yes or no), handwashing before consuming food (always, sometimes, never), diarrhea within 15 days of fecal collection (yes or no), stomach pain within 15 days of fecal collection (yes or no), nausea within 15 days of fecal collection (yes or no), vomiting within 15 days of fecal collection (yes or no) (Supplementary Table 1).

Logistic regression analysis was used to identify factors associated with *Blastocystis* infection using demographic and socioeconomic variables with R version 3.5.1 (R Core Team 2018). *P*-values < 0.05 were considered statistically significant.

## Results

### *Blastocystis* prevalence and subtypes using NGS

Of the 98 samples from rural schoolchildren included in this study, 72.4% were *Blastocystis* positive via PCR and NGS sequencing. Prevalence and subtypes of *Blastocystis* by location, gender, and age are presented in Table 1. Samples were collected from three schools in Apulo, Department of Cundinamarca, Colombia, and a higher prevalence was observed among samples from Pantanos with 84.4% of samples being *Blastocystis-*positive (27/32) followed by a 66.7% from Naranjal (6/9) and Naranjalito (38/57). Children ranged in age from 4 to 16 years, and for ages with more than one sample tested, *Blastocystis* prevalence ranged from 33.3% in 14-year-old to 90.9% in 6-year-old. *Blastocystis* was more common in females with 77.1% (37/48) testing positive, while 68.0% (34/50) of males were positive.

**Table 1:** Number of children examined, number of children *Blastocystis*-positive, prevalence (%), and subtypes of *Blastocystis* by location, gender, and age

		No. of samples examined	No. of positive samples	Prevalence (%)	*Blastocystis* subtypes (No. of Samples)
School	Naranjal	9	6	66.7	ST1 (1), ST2 (1), ST3 (2), ST1/ST2 (1), ST1/ST3 (1)
	Pantanos	32	27	84.4	ST1 (5), ST2 (6), ST3 (7), ST1/ST2 (2), ST1/ST3 (5), ST1/ST2/ST3 (1), ST2/ST3/ST4 (1)
	Naranjalito	57	38	66.7	ST1 (7), ST2 (16), ST3 (6), ST1/ST2 (1), ST1/ST3 (4), ST2/ST5 (1), ST3/ST5 (3)
Gender	Male	50	34	68.0	ST1 (8), ST2 (9), ST3 (9), ST1/ST3 (5), ST1/ST2/ST3 (1), ST2/ST3/ST4 (1), ST3/ST5 (1)
	Female	48	37	77.1	ST1 (5), ST2 (14), ST3 (6), ST1/ST2 (4), ST1/ST3 (5), ST2/ST5 (1), ST3/ST5 (2)
Age	4	1	1	100	ST1 (1)
	5	2	1	50.0	ST2 (1)
	6	11	10	90.9	ST1 (2), ST2 (3), ST3 (2), ST1/ST3 (2), ST3/ST5 (1)
	7	14	10	71.4	ST1 (1), ST2 (4), ST3 (2), ST1/ST2 (1), ST1/ST3 (2)
	8	9	5	55.6	ST1 (1), ST2 (1), ST3 (2), ST2/ST5 (1)
	9	11	8	72.7	ST1 (1), ST2 (4), ST1/ST2 (2), ST1/ST3 (1)
	10	16	13	81.3	ST1 (2), ST2 (4), ST3 (5), ST1/ST3 (2)
	11	10	8	80.0	ST1 (1), ST2 (1), ST3 (2), ST3/ST5 (2), ST1/ST2/ST3 (1), ST2/ST3/ST4 (1)
	12	11	7	63.6	ST1 (1), ST2 (4), ST1/ST2 (1), ST1/ST3 (1)
	13	5	4	80.0	ST1 (2), ST2 (1), ST1/ST3 (1)
	14	6	2	33.3	ST1 (1), ST1/ST3 (1)
	15	1	1	100	ST3 (1)
	16	1	1	100	ST3 (1)

Five *Blastocystis* subtypes (ST1-ST5) were detected in this study (Tables 1 and 2). ST1, ST2, and ST3 were identified as mono-subtype or mixed subtype infections, while ST4 and ST5 were only detected as mixed subtype infections. ST4 was only detected in a boy attending the Pantanos school in combination with ST2 and ST3 (Table 1). ST5 was detected in four children attending the Naranjalito school (three girls and one boy) in two combinations, ST3/ST5 in three children and ST2/ST5 in one child.

**Table 2:** *Blastocystis* frequency for each subtype (ST) in mono-infections and for the different combinations in mixed infections determined using next-generation amplicon sequencing

	*Blastocystis* mono ST infections			Total *Blastocystis* mono ST infections	*Blastocystis* mixed ST infections						Total *Blastocystis* mixed ST infections
	ST1 only	ST2 only	ST3 only		ST1/ST2	ST1/ST3	ST2/ST5	ST3/ST5	ST1/ST2/ST3	ST2/ST3/ST4	
Total number of positive samples	13	23	15	51	4	10	1	3	1	1	20
Percentage of all samples	13.3	23.5	15.3	52.0	4.1	10.2	1.0	3.1	1.0	1.0	20.4
Percentage of positive samples	18.3	32.4	21.1	71.8	5.6	14.1	1.4	4.2	1.4	1.4	28.2

Mono-subtype infections were more common than mixed infections representing 71.8% (51/71) and 28.2% (20/71) of the *Blastocystis*-positive samples, respectively (Table 2; Supplementary Table 2). ST2 and ST3 were the most frequently observed subtypes in this population, and each was found in 42.3% (30/71) of the *Blastocystis*-positive samples either as mono-infections or mixed infections (Table 2). ST2 was more commonly observed as a mono-infection (*n* = 23) compared to mixed infection (*n* = 7), while ST3 was observed in equal proportions as a mono (*n* = 15) or mixed infection (*n* = 15) (Table 2). Subtype 1 was observed in 39.4% (*n* = 28) of *Blastocystis*-positive samples, as either mono (*n* = 13) or mixed-infections (*n* = 15). ST4 and ST5 were less common among the study participants representing only 1.4% (*n* = 1) and 5.6% (*n* = 4) of *Blastocystis*-positive samples, respectively. Both ST4 and ST5 were only identified as part of mixed infection combinations. Mixed ST1/ST3 infection was the most common subtype combination and was found in 50% (*n* = 10) of the samples containing multiple subtypes. Mixed subtype infections with combinations of ST1/ST2, ST2/ST5, and ST3/ST5 were found in 4, 1, and 3 samples, respectively. Two samples were observed to contain three subtypes with combinations of ST1/ST2/ST3 and ST2/ST3/ST4 observed in one sample each.

### Intra-subtype variability

Fifty-five unique OTUs were detected among the *Blastocystis* subtypes present in this study (Table 3). Among the three subtypes more commonly identified in this study (ST1, ST2, and ST3), ST2 had the highest intra-subtype variability with 23 unique variants among 30 ST2-positive samples followed by ST1 with 18 unique variants among the 28 ST1-positive samples. Subtype 3 displayed the least intra-subtype diversity with only 9 unique variants among 30 ST3-positive samples. Subtype 5 was the only subtype present in multiple samples for which all variants were observed in only one sample each (Table 3).

**Table 3:** Information on *Blastocystis* intra-subtype variants obtained by next-generation amplicon sequencing

Subtype	No. of unique ST variants	No. of samples containing variant	GenBank Accession number
ST1	18	3	OP725928
		4	OP725930
		2	OP725931
		4	OP725934
		2	OP725935
		1	OP725940
		4	OP725941
		2	OP725943
		1	OP725947
		1	OP725948
		1	OP725949
		3	OP725952
		1	OP725964
		1	OP725967
		1	OP725969
		2	OP725971
		1	OP725972
		1	OP725975
ST2	23	6	OP725926
		6	OP725927
		5	OP725929
		3	OP725932
		4	OP725936
		4	OP725937
		4	OP725942
		2	OP725944
		1	OP725950
		3	OP725951
		1	OP725954
		1	OP725955
		1	OP725956
		2	OP725958
		1	OP725959
		1	OP725960
		1	OP725961
		1	OP725962
		2	OP725963
		1	OP725965
		1	OP725966
		1	OP725973
		1	OP725976
ST3	9	15	OP725923
		3	OP725924
		4	OP725925
		1	OP725933
		2	OP725938
		2	OP725939
		1	OP725945
		1	OP725946
		1	OP725953
ST4	1	1	OP725970
ST5	4	1	OP725957
		1	OP725968
		1	OP725974
		1	OP725977

Samples frequently contained two unique variants of ST1 and ST2 (see Supplementary Table 2), and up to three unique variants of ST2 were detected in two samples (T62 and T80). However, multiple variants of ST3, ST4, or ST5 were not observed in the same sample. Furthermore, while unique variants of ST1 and ST2 were relatively evenly distributed among individual samples, one unique variant of ST3 was predominant in this population and was observed in 15 of 30 *Blastocystis* ST3-positive samples (Table 3).

### Blastocystis subtypes and intra-subtype variability among siblings living in the same household

**Fig. 1:**
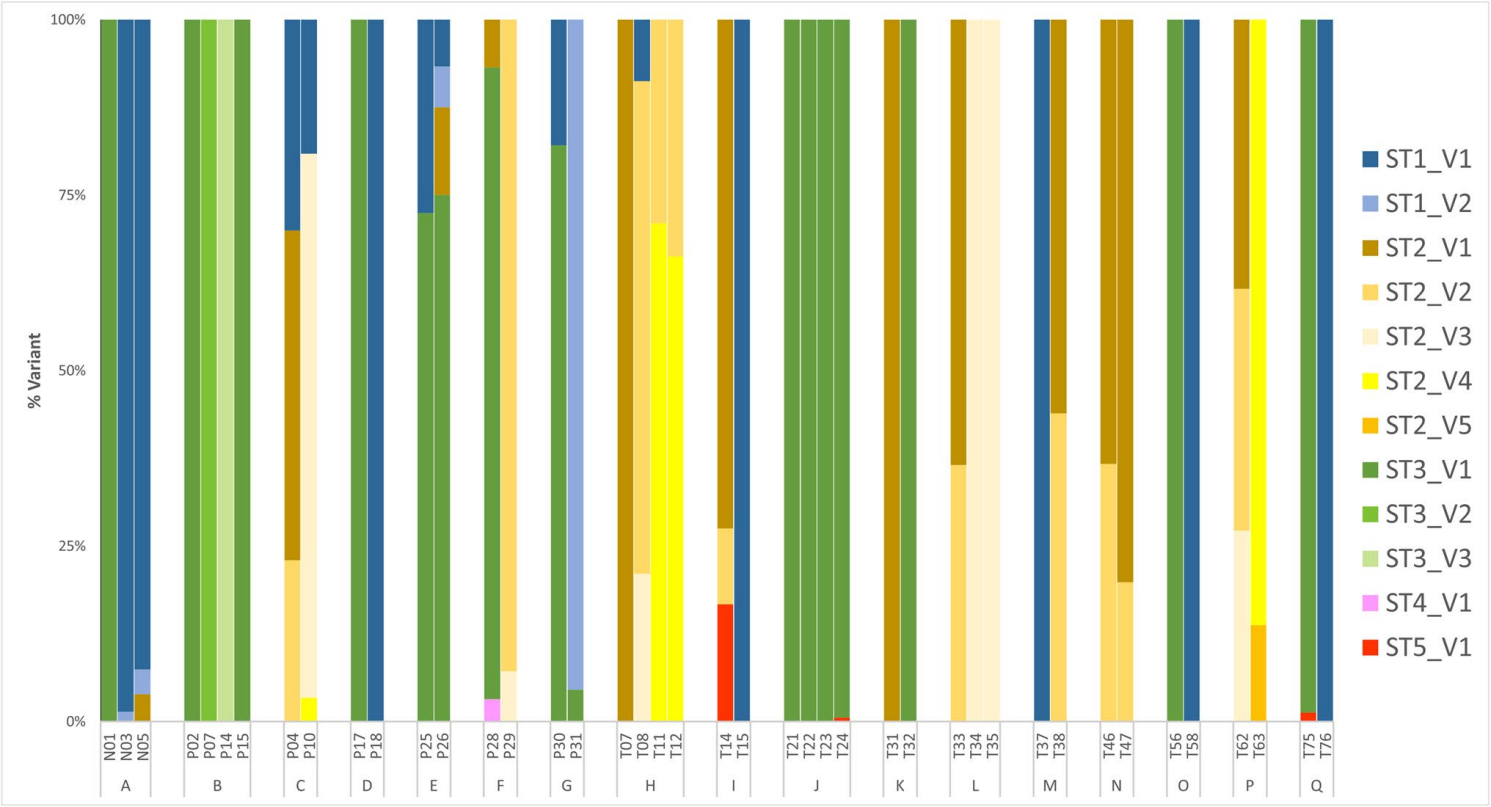
Comparison of *Blastocystis* subtypes and sequence variants among children living within the same household. There were 17 households with multiple *Blastocystis* positive children in the household. These households are represented by letters A to Q. Variants within each subtype are not comparable between households and only represent within household sequence variants. For detailed information on sequence variants, see Supplementary Table 2

There were 25 households that had at least two children participating in the study. In three of those 25 households, all children were negative, in five households, only one sibling was *Blastocytis* positive, and in the other 17 households, all children were positive (42 children). When comparing subtypes among *Blastocystis* positive children from the same household, perfect agreement among siblings was rare. In fact, only one household had siblings that shared identical ST profiles; household N had two siblings that were both positive for the same sequence variants of ST2 (Fig. 1). It was more commonly observed that while some siblings within the same house shared the same sequence variants, non-shared variants were also present. This was the case for eight households (A, B, C, E, G, H, J, and L). In the remaining eight households (D, F, I, K, M, O, P, and Q), no overlap in sequence variants was observed among siblings. Only four households (B, L, N, and P) had a single ST present. Notably, multiple sequence variants of the same ST were commonly observed among siblings with up to two variants of ST1 in the same household (A), up to five variants of ST2 in the same household (P), and up to three variants of ST3 in the same household (B).

### Association between sociodemographic variables and presence of Blastocystis

Logistic regression analyses were performed to determine if any associations existed between *Blastocystis* infection and gender, age, school, monthly income, number of people per household, type of flooring, aqueduct service, toilet location, dogs in the house, cats in the house, chickens in the house, handwashing before consuming food, and diarrhea, stomach pain, nausea, or vomiting within 15 days prior sampling (Supplementary Table 1). Among these variables, gender, age, number of people per household, toilet location, dogs in the house, cats in the house, chickens in the house, handwashing before consuming food, and nausea within 15 days prior sampling were all found to have statistically significant associations with one or more of the following outcomes: *Blastocystis* infection (any subtype or subtype combination in an individual sample), ST1 infection, ST2 infection, ST3 infection, or mixed STs infection (the presence of more than one subtype in an individual sample) (Table 4).

**Table 4:** Sociodemographic variables studied by logistic regression analysis that showed statistically significant association with *Blastocystis* infection (any subtype or subtype combination), single subtype infection, or mixed subtype infections

	Variable	Log odds	*p*-value	95% CI
*Blastocystis*	Chickens in house	-1.83	0.04	-3.5, -0.1
	Hand washing (never)	3.77	0.04	0.2,7.3
ST1	Dogs in house	3.13	0.03	0.4, 5.9
	Cats in house	1.62	0.02	0.3, 3.0
	Chickens in house	-2.07	0.02	-3.7, -0.4
ST2	Persons per house (≥ 7)	4.00	0.01	0.9, 7.1
	Chickens in house	-1.65	0.04	-3.2, -0.1
	Nausea within 15 days prior sampling	-2.12	0.04	-4.1, -0.1
ST3	Age (years)	-0.17	0.03	-0.3, 0.0
	Toilet location (outside)	2.46	0.02	0.5, 4.5
Mixed STs	Gender (male)	-2.01	0.04	-3.9, -0.1
	Toilet location (outside)	3.36	0.03	0.4, 6.3
	Cats in house	2.54	0.01	0.6, 4.5
	Chickens in house	-3.89	0.002	-6.3, -1.4
	Hand washing (never)	5.88	0.01	1.3, 10.4

*CI* confidence intervals

Only two variables had significant associations with *Blastocystis* infection: the presence of chickens in the house and handwashing before consuming food (Table 4). The odds of having *Blastocystis* was lower when chickens were present in the house (OR: -1.83, 95% CI: -3.5 to -0.1, *p* = 0.04) and higher among participant who reported never washing their hands before consuming food (OR: 3.77, 95% CI: 0.2 to 7.3, *p* = 0.04).

Among study participants with ST1 infection, only variables related to the presence of animals in the house were significantly associated with infection risk. Both the presence of cats and the presence of dogs increased the odds of ST1 infection (cats, OR: 1.62, 95% CI: 0.3 to 3.0, *p* = 0.02; dogs, OR: 3.13, 95% CI: 0.4 to 5.9, *p* = 0.03) while the presence of chickens was associated with lower odds of ST1 infection (OR: -2.07, 95% CI: -3.7 to -0.4, *p* = 0.02). ST2 infection was significantly associated with three variables. The odds of ST2 infection were higher among children in households of ≥ 7 persons (OR: 4.00, 95% CI: 0.9 to 7.1, *p* = 0.01) but lower among those with chickens in the house (OR: -1.65, 95% CI: -3.2 to -0.1, *p* = 0.04) and who reported experiencing nausea within the last 15 days (OR: -2.12, 95% CI: -4.1 to -0.1, *p* = 0.04). Age and toilet location were significantly associated with ST3 infection. Age had a negative association with ST3 infection with a -0.17 reduction in infection risk for each year of increase in age (OR: -0.17, 95% CI: -0.3 to 0.0, *p* = 0.03), while having a toilet location outside of the home was associated with an increase in ST3 infection risk (OR: 2.46, 95% CI: 0.5 to 4.5, *p* = 0.02).

Mixed ST infection had significant associations with five variables, gender, toilet location, cats in the house, chickens in the house, and handwashing. Reporting gender as male (OR: -2.01, 95% CI: -3.9 to -0.1, *p* = 0.04) and the presence of chickens in the house (OR: -3.89, 95% CI: -6.3 to -1.4, *p* = 0.002) were both associated with lower odds of mixed infection, while having a toilet outside the house (OR: 3.36, 95% CI: 0.4 to 6.3, *p* = 0.02), cats in the house (OR: 2.54, 95% CI: 0.6 to 4.5, *p* = 0.01), and never washing hands before consuming food (OR: 5.88, 95% CI: 1.3 to 10.4, *p* = 0.01) all increased the odds of mixed infection. No other statistically significant associations between *Blastocystis* infection status or individual subtypes were found.

## Discussion

*Blastocystis* is common in humans worldwide, and at least 14 subtypes have been reported in humans to date (Alfellani et al. 2013; Jinatham et al. 2021; Khaled et al. 2021, 2020; Jinatham et al. 2021; Osorio-Pulgarin et al. 2021; Ramírez et al. 2016). It is likely that the genetic diversity between or even within subtypes could be a factor influencing infection outcomes in human hosts including the presence and type of symptoms experienced during infection. However, studies seeking to define such relationships are lacking. Determining a link between subtype and symptomology is a difficult task which is further complicated by the ability of individuals to be host to multiple subtypes at the same time. These instances of mixed infection are not well characterized in humans, in part due to the technical difficulties associated with defining individual subtype sequences from among complex mixtures of parasite DNA. In the present study, an NGS method capable of discriminating mixed infections was used to measure the prevalence and subtype diversity of *Blastocystis* among school aged children from a rural region of Colombia and associations between infection status and socioeconomic and demographic factors were analyzed via logistic regression analysis.

*Blastocystis* prevalence was high among the study participants with 72.4% of the 98 participants found positive by PCR and NGS. This observation is concordant with other molecular surveys of *Blastocystis* from Colombia where infection rates among children were generally high (Londoño-Franco et al. 2014; Sánchez et al. 2017; Bryan et al. 2020; Higuera et al. 2020; Osorio-Pulgarin et al. 2021). The prevalence of *Blastocystis* among children in this study is also similar to a recent study among children from a rural community in the neighboring country of Panama which reported a prevalence of 74.2% in that population (Perea et al. 2020). However, the prevalence observed among Colombian children is higher than many other studies from children from around the world. Only 2.6% of children were *Blastocystis* positive in a study from the USA (Scanlan et al. 2016). A study using similar methods in a rural population in Mexico observed 43.9% of children were *Blastocystis* positive (Rojas-Velázquez et al. 2019). A large-scale study from France reported a prevalence of 26.3% among children. (El Safadi et al. 2016). In a study of *Blastocystis* in children from six countries (Azerbaijan, Czechia, Jordan, Nigeria, Sudan, and Tanzania), prevalence was found to range from 15% in Czechia up to 55% in Nigeria (Cinek et al. 2021). Conversely, the prevalence of *Blastocystis* in this study does not reach the prevalence of 100% which was reported in Senegalese children (El Safadi et al. 2014). Clearly, *Blastocystis* is common in children and adults around the world, but the factors influencing the variability in prevalence from different populations are not well defined and should be explored in future studies of *Blastocystis*.

An NGS strategy was used to determine subtype presence and diversity in PCR positive samples in this study. There were five subtypes (ST1-ST5) present among the 71 *Blastocystis*-positive children. Of these subtypes, ST1-ST3 were most commonly present in *Blastocystis*-positive samples while ST4 and ST5 were occasional findings and always present as mixed ST infections (Tables 1 and 2). These findings agree with other studies reporting *Blastocystis* subtypes in humans from Colombia (Ramírez et al. 2014, 2017; Sánchez al. 2017; Espinosa et al. 2018; Higuera et al. 2020; Hernández et al. 2021; Osorio-Pulgarin et al. 2021). Indeed, these trends are true for humans worldwide, although some reports of ST4 being more common in humans from Europe are present in the literature (Alfellani et al. 2013; El Safadi et al. 2016). As most subtypes are not known to be restricted to an individual host or location, the factors influencing differences in subtype prevalence among different hosts and regions of the world remain to be described.

Previous studies employing NGS to study *Blastocystis* in humans and other animals have demonstrated that this strategy is well suited to exploring inter and intra subtype diversity within individual samples (Maloney et al. 2019, 2021a; Rojas-Velázquez et al. 2019; Vega et al. 2021). In the present study, mixed infections were common, with 28.2% of *Blastocystis* positive samples containing two or more subtypes. The prevalence of mixed infections in this study is within the range reported from other studies which used NGS for subtype identification. In humans from Turkey and Mexico, mixed infections represented 20.6% and 13.7% of positive samples, respectively (Rojas-Velázquez et al. 2019; Sarzhanov et al. 2021). However, a study analyzing the co-occurrence of *C. difficile and Blastocystis* in humans from Colombia observed nearly equal proportions of mono (48.4%) and mixed subtype infections (51.6%) (Vega et al. 2021). Interestingly, as in the present study, ST5 was only observed in mixed infections. These findings highlight that mixed subtype infections are common and should be considered in studies aiming to explore the relationship between *Blastocystis* infections and health and disease. Similarly, studies assessing the prevalence of subtypes in a given host or region should attempt to describe the full extent of diversity in each sample to better understand the epidemiology of *Blastocystis*.

Intra-subtype diversity is well recognized for *Blastocystis* and can be up to 3% for individual subtypes (Stensvold and Clark 2020). This genetic diversity within subtypes could also be a factor influencing differences in host specificity and pathogenicity. Intra-subtype diversity was quite high in the present study with 55 unique genetic variants observed among the five subtypes present (Table 3). All subtypes which were present in more than one sample had multiple variants, and intra-subtype diversity was frequently observed within individual samples (Table 3 and Supplementary Table 2). Interestingly, although ST1, ST2, and ST3 were present in similar proportions among the study participants, ST3 exhibited far less intra-subtype diversity than ST1 or ST2. This difference in diversity was largely due to a single variant of ST3 being far more common than any other ST3 variants in this study while variants of ST1 and ST2 were relatively evenly distributed. The same observation was made among the variants of ST1, ST2, and ST3 from human studies from Mexico and Turkey (Rojas-Velázquez et al. 2019; Sarzhanov et al. 2021). The consistent observation of less diversity in ST3 and more in ST1 and ST2 could support the idea that ST3 may have a common source of transmission in human populations while other subtypes may be more variable in their transmission pathways. Another potential explanation could be that ST3 represents a more recent acquisition in humans and thus is less diverse that other subtypes which may have been circulating in human populations for longer time periods. Such conclusions would require further research and validation. The high degree of variability for all subtypes observed in this study indicates that describing associations between either individual subtypes or subtype variants will be difficult using the current classification system for *Blastocystis* identification.

Among the study participants, there were 42 children who could be segregated into 17 households with more than one *Blastocystis* positive child living in the same household. Comparisons among siblings living in these 17 households demonstrated that only one household (N) had siblings that shared the exact same sequence variant profile (Fig. 1). Furthermore, while overlap in sequence variants among siblings was common, subtype and sequence variant diversity within households was also frequently observed. This suggests that there are likely multiple infection sources for the children participating in this study, and that not all of those infection sources are shared within families. Genetic diversity of *Blastocystis* among family members has been previously studied using a PCR/Sanger approach with no subtype variant information (Nagel et al. 2012; Scanlan et al. 2016; Jinatham et al. 2021). A study that included 11 symptomatic *Blastocystis*-positive patients and 17 of their family members found that 16 of the 17 household contacts had the same subtype as their symptomatic family member (Nagel et al. 2012). However, a study in the USA that included 50 healthy family units, total of 139 individuals, did not find any *Blastocystis*-positives among the families of the 10 individuals that were found *Blastocystis*-positive suggesting that human–human transmission was improbable within families that took part in the study (Scanlan et al. 2016). Similarly, in a study that included six family units in a rural community of northern Thailand, it was found that in the three households with two *Blastocystis*-positive family members, both occupants carried different subtypes (Jinatham et al. 2021). More studies that include subtyping and analysis of intra-subtype variability within family units are required to further understand *Blastocystis* transmission dynamics within families. By collecting samples from animals living in close contact with family units, it would also be possible to better assess the importance of zoonotic transmission of *Blastocystis* between families and their animal contacts.

The collection of demographic and socioeconomic data in conjunction with sample collection allowed for the assessment of associations between these variables and *Blastocystis* infection risk. Because ST1-ST3 and mixed infections were all common among the study participants, associations between these infection types and explanatory variables were also assessed. However, because ST4, ST5, and individual variants were not abundant among study participants, these outcomes were not assessed. Logistic regression analysis found significant associations for nine variables (Table 4). Among these variables, number of people per household, toilet location, handwashing before consuming food, dogs in the house, and cats in the house were all found to increase risk for at least one of the tested infection outcomes, while male gender, age, chickens in the house, and nausea within 15 days prior sampling were associated with decreased infection risk for at least one of the tested outcomes.

The association between *Blastocystis* infection in humans and many of the variables with significant outcomes, such as age, gender, number of people per household, toilet location, and handwashing before consuming food provide valuable insight into potential factors influencing infection risk. However, such findings are not particularly surprising given that associations with similar variables related to demographics, socioeconomic status, and hygienic behavior have been observed previously (Rojas-Velázquez et al. 2019; Paulos et al. 2018; Abdulsalam et al. 2013; Salazar-Sánchez et al. 2020). The significant associations between infection status and symptoms and animal exposure are particularly relevant as the pathogenicity and zoonotic transmission of *Blastocystis* are topics of interest and debate.

In this study, the only variable related to gastrointestinal symptoms with a significant association with infection was nausea within 15 days prior sampling (Table 4). Experiencing nausea within 15 days prior sampling reduced the risk of ST2 infection among study participants. Interestingly, a recent study from Mexico reported a significant association between experiencing gastrointestinal symptoms and a reduction in infection risk for ST3 (Rojas-Velázquez et al. 2019). Associations between lower odds of infection and recent intestinal symptoms while intriguing are not easily explained, especially in the context of conflicting reports of positive associations between infection and symptoms such as a recent study in children from Panama which reported a significant association between *Blastocystis* infection and diarrhea (Perea et al. 2020). Many studies report *Blastocystis* as causing no symptoms indicating that it may be a common organism in the intestine of healthy humans (Deng et al. 2021). The association between infection status and symptoms was only observed at the subtype level in this study. Our findings may indicate that exploring such relationships may need to happen at a more refined level to better explain potential associations between infection and symptoms.

The significant associations between infection and animal contact were remarkably common among children in this study. There were seven significant associations, and of the five infection outcomes tested (*Blastocystis* infection, ST1 infection, ST2 infection, ST3infection, and mixed infection), only ST3 infection had no significant association with animal contact (Table 4). The presence of cats in the house and dogs in the house both had associations with increased infection risk with ST1 being associated with cats and dogs and mixed infections with only cats. A similar finding was recently reported from a study in Peru which found a significant association between *Blastocystis* and dogs (Salazar-Sánchez et al. 2020). Notably, *Blastocystis* infection in cats and dogs may be uncommon as it has been reported that these animals often have low infection rates, so why contact with these animals would increase infection risk among the study population is unclear (Paulos et al. 2018; Rudzińska et al. 2022). However, sampling was not performed for animals in contact with the participants of this study, so whether infection risk is related to direct animal to human transmission or to other factors associated with pet ownership or the behavior of children could not be assessed. The common association between *Blastocystis* infection and pet ownership in this study indicates further exploration of zoonotic transmission between humans, especially children, and pets is warranted.

A rather surprising finding in this study was the negative association between contact with chickens and infection. Reporting chickens in the house was associated with a lower risk of infection for *Blastocystis* (any subtype or combination), ST1 infection, ST2 infection, and mixed infection (Table 4). Such an observation seems counterintuitive to concepts of transmission dynamics among humans and animals and the conditions surrounding the presence of chickens in a domestic setting. Yet the association was consistently observed for all infection outcomes but ST3 infection in this study. Furthermore, subtypes which are common in chickens, such as ST6 and ST7, were not observed among the participants of this study (Maloney et al. 2021b). Perhaps contact with chickens is capturing some other socioeconomic or demographic variable not measured in this study. For example, a higher occurrence of *Blastocystis* in urban areas has been reported in a study from Spain, indicating lifestyle differences could impact infection status in humans (Paulos et al. 2018). Although all participants of the study live in an area considered rural, reporting close contact with chickens may be capturing a segment of the population with lifestyle differences which reduce their risk of *Blastocystis* infection. Studies designed to interrogate this relationship more thoroughly would be needed to demonstrate what may be driving a potential association between chickens and infection risk.

## Conclusions

The relationship between *Blastocystis* and human health and disease is topic of much research interest. *Blastocystis* subtypes likely exhibit differences in their ability to cause infection and disease among humans and other animals. Yet, the relationships between *Blastocystis* genetic variability within individual hosts and host species and the role of this variability in influencing infection outcomes are both complex and understudied. By using a targeted massively parallel sequencing strategy paired with demographic data, this study provides foundational data on subtype relationships and mixed infection and infection risk. As the *Blastocystis* community continues to seek answers to questions related to transmission, pathogenicity, and host specificity, these types of studies are essential to produce the data needed to clarify these relationships.

## Supplementary Information

Below is the link to the electronic supplementary material.

Supplementary file1 (XLSX 20 KB)

Supplementary file2 (XLSX 18 KB)

## Data Availability

All relevant data are within the article and its additional files. Raw FASTQ files were submitted to NCBI’s sequence read archive under project PRJNA896134 and accession numbers SRR22105641- SRR22105683. The nucleotide sequences obtained in this study have been deposited in GenBank under the accession numbers OP725923-OP725977.
